# Multi-omics integrated analysis reveals the impact of cytokinin on sex differentiation in industrial hemp

**DOI:** 10.1093/aobpla/plaf019

**Published:** 2025-03-31

**Authors:** Yushu Chen, Mengdi Yu, Junbao Zhang, Xuesong Wang, Qingyi Shao, Sen Yang, Jiaang Cao, Shanshan Li, Lijie Liu

**Affiliations:** College of Life Science and Agriculture Forestry, Qiqihar University, 42 Wenhua street, Qiqihar 161006, Heilongjiang, China; College of Life Science and Agriculture Forestry, Qiqihar University, 42 Wenhua street, Qiqihar 161006, Heilongjiang, China; College of Life Science and Agriculture Forestry, Qiqihar University, 42 Wenhua street, Qiqihar 161006, Heilongjiang, China; College of Life Science and Agriculture Forestry, Qiqihar University, 42 Wenhua street, Qiqihar 161006, Heilongjiang, China; College of Life Science and Agriculture Forestry, Qiqihar University, 42 Wenhua street, Qiqihar 161006, Heilongjiang, China; College of Life Science and Agriculture Forestry, Qiqihar University, 42 Wenhua street, Qiqihar 161006, Heilongjiang, China; College of Life Science and Agriculture Forestry, Qiqihar University, 42 Wenhua street, Qiqihar 161006, Heilongjiang, China; College of Life Science and Agriculture Forestry, Qiqihar University, 42 Wenhua street, Qiqihar 161006, Heilongjiang, China; College of Life Science and Agriculture Forestry, Qiqihar University, 42 Wenhua street, Qiqihar 161006, Heilongjiang, China

**Keywords:** industrial hemp, cytokinin, combined multi-omics analysis, sex differentiation

## Abstract

To increase the cannabidiol (CBD) content of industrial hemp, male hemp was screened out by spraying cytokinin at the three-leaf stage of seedlings, and more female hemp was cultivated. 6-BA 60 mg·L^−1^ treated female flowers of industrial hemp were subjected to transcriptomic, proteomic, and metabolomic analyses to investigate the changes and molecular mechanisms of gene expression and metabolites and related pathways of 6-BA in the development of female flowers of industrial hemp. The results showed that 1189 differentially expressed genes (DEGs), 168 differentially expressed proteins (DEPs), and 138 DAMs were screened compared with the control. Functional enrichment analysis revealed that phytohormone signaling, starch and sucrose metabolism, flavonoid biosynthesis, phenylpropane metabolism, and glutathione metabolism were the major pathways enriched, and differential genes, proteins, and metabolites enriched in the above pathways were further followed up and analyzed. It was found that, among them, *CCL1*, *PAL1,* and C4H were the key genes and proteins involved in the phenylpropane metabolic pathway, *CYP450* and *FLS* were not only the upstream genes in the flavonoid biosynthesis pathway, but *CYP450* were also involved in the synthesis of phytohormones and catabolism. *FLS* was related to the synthesis of saccharides. It was hypothesized that the carbohydrates might synergistically act with cytokinins to induce female flower differentiation in industrial hemp. The flavonoid biosynthesis pathway and glutathione metabolism pathway are also closely related to feminization. This paper provides a reference for subsequent studies on sex differentiation in hemp or other plants.

## Introduction

Hemp (*Cannabis sativa L*.), also known as fire hemp, line hemp, wild hemp, etc., is annual herbaceous plant of Moraceae (*Cannabis Linn.*), and it is native to central Asia. Because of its rapid growth and adaptability, and it is now widely distributed around the world (Bonini et al. 2018). Hemp contains a variety of chemical components, currently isolated and identified compounds have more than 560 kinds of compounds, including cannabinoids. It is now found that cannabinoids are mostly accumulated in the lumen of the glandular hairs of female plants (Elsohly et al. 2017). Cannabinoids have abundant pharmacological activities, such as analgesic, anti-inflammatory, antiepileptic, anticonvulsant, immunomodulation, etc., of which tetrahydrocannabinol (THC) and cannabidiol (CBD) are the main ones (Whiting et al. 2015). THC is addictive, and the hemp varieties that do not have addictive properties with a content of THC less than 0.3% in China are called industrial hemp (Rupasinghe et al. 2020), CBD can inhibit THC-induced anxiety, depression, tachycardia, and other adverse effects, enhance and prolong the efficacy and duration of action of THC (Pellati et al. 2018; Nichols and Kaplan 2020) and has high utilization value in the medical, beauty, and food fields. The CBD content of female industrial hemp plants is much higher than that of male plants, but the CBD content of female plants decreases dramatically after pollination. Therefore, to obtain more CBD, it is necessary to breed more female plants or convert more male plants to female plants.

Sex differentiation is prevalent and is an important symbol of the transition from nutritive to reproductive growth in plants. Plant sex differentiation is mainly influenced by sex-determining genes, sex chromosomes, environmental factors such as temperature, photoperiod, minerals, and many other factors (Andrés and Coupland 2012) and also regulated by endogenous hormone signals, such as growth hormone, brassinolide, cytokinin, ethylene, gibberellin, and methyl jasmonate (Louis and Durand 1978; Chailakhyan 1979; Durand and Durand 1984; Irish and Nelson 1989; Chen et al. 2017). It was found that some hormones can effectively promote the development of plant females, such as spinach seedlings with roots immersed in 15 mg/L indoleacetic acid for a week, were found that the ratio of male to female increased (Renner and Hyosig 2001), a much higher number of female flowers than the number of male flowers were produced in bitter gourd with foliar spraying of 150 mg/L naphthaleneacetic acid (Khatoon and Moniruzzaman 2019), and spraying the appropriate concentration of naphthaleneacetic acid can make the female flowers of bitter gourd bloom earlier (Zou, Han and Sun 2017). There is a significant difference in the content of auxin between male and female hemp, and the auxin content of female hemp is 30 times higher than that of male hemp; similarly, in female and monoecious lines of cucumber, the auxin content of female lines is higher (Galun, Izhar and Atsmon 1965; Rudich, Halevy and Kedar 1972). Cytokinins promote plant tissue differentiation and growth and have the effect of promoting female flower development (Jaligot et al. 2011), female flowers were increased when plant (*Sapium sebiferum* L.) treatd by 6-BA and the genes related to gynoecium development were significantly up-regulated, while the genes related to the development of the male organs were down-regulated, suggesting that cytokinins can regulate the floral sex differentiation by directly targeting the genes related to the floral organs (Ni et al. 2018), the number of flowers and the ratio of male to female flowers were significantly increased when the inflorescence meristematic tissues of (*Jatropha curcas* L.) treated with exogenous spraying of 6-BA (Pan et al. 2014), and many studies have shown that cytokinins can induce the differentiation of female flowers in hemp (Chailakhyan and Khryanin 1978; Petit et al. 2020; Flajsman, Slapnik and Murovec 2021).

In the metabolic process of plants, there are differences between their male and female individuals. This difference may lead to difference of some physiological and biochemical indexes of plants, such as enzyme activity, secondary metabolites, and endogenous hormone content levels, etc., moreover, it is mainly manifested in redox capacity, male individuals are in the state of relative oxidation, while female individuals are in the state of relative reduction (Cao 1965), so some physiological and biochemical indexes can be tested to identify the sex of plants. Enzymes are one of the direct products of gene expression, so the differencein enzyme activity can also reflect the difference between female and male plants to acertain extent, such as the sex difference in spinach is related to the number of enzyme bands of peroxidase (Penel and Greppin 1972). And, it was found that the ginkgo plants had a smaller decrease in the net photosynthesis rate, superoxide dismutase (SOD), and catalase (CAT) activities of the female plants, and peroxidase enzyme (POD) activity, glutathione content were high and hydrogen peroxide and malondialdehyde content increased less compared with male ginkgo strains in the process of aging (Shi et al. 2012), and proline content related to anther development was much higher in Ginkgo males than in females (Zhao and Tian 1993). The soluble sugar content of juniper female inflorescences is significantly higher than male inflorescences in most periods, probably because carbohydrates are one of the important factors affecting sex differentiation (DeSoto, Olano and Rozas 2016), and the soluble protein content of polygynous plants in chestnut is significantly higher than that of oligogynous plants, indicating that the accumulation of soluble proteins is an important material basis for the formation of female flowers (Zhou 2021).

Regarding hemp sex differentiation, screened genes and transcription factors involved in flowering time and sex determination in hemp have been screened by Jordi Petit using a GWAS approach, and the genetic mechanisim associatied with sex differentiation traits in hemp was further calrified (Petit et al. 2020), and male flowers on the female plant could beeasily and consistently produced when spray application of silver thiosulfate on hemp leaves under short-day conditions (Lubell and Brand 2018), spraying hemp plants with colloidal silver showed the effect of inducing male flowers on female plants, producing up to 379 male flowers on each hemp plant, and the viability and fertility of the induced male flowers were confirmed by pollen grain fluorescein diacetate (FDA) staining, pollen isolation and *in vivo* germination assays, counting of the number of seeds that developed after crossing, and evaluation of germination rates of the developed seeds, which showed that the progeny had a higher and more uniform ratio of total CBD to total THC (Flajsman, Slapnik and Murovec 2021). Currently, transcriptome (Guerriero et al. 2017; Braich et al. 2019), proteome (Cheng et al. 2023), metabolome (Happyana et al. 2013), and combined multi-omics analysis (Onofri, de Meijer and Mandolino 2015) have been used in hemp research, but less in the field of hemp sex differentiation, especially multi-omics joint analysis, and the relationship and mechanism of gene expression, metabolites, and related pathways in hemp with its sex differentiation are still unclear. The purpose of this paper is to increase the content of cannabinoid CBD in industrial hemp, using the method of spraying cytokinin (6-BA) at the three-leaf stage of seedlings to cultivate more female hemp, we combined transcriptomic, proteomic, and metabolomic analyses, to investigate the changes in gene expression, metabolites, and related pathways in the process of 6-BA-regulated sex differentiation of industrial hemp and reveal the molecular mechanisms.

## Materials and methods

### Plant materials

Full-grain industrial hemp seeds (provided by the Ministry of Education Engineering Research Center for Cold Zone Hemp and Products, Qiqihar University) were selected and germinate at room temperature 25 °C under natural light, when seedlings grow to the three-leaf stage, the plant hormone 6-BA (the concentration is 15, 30, 60, 120 mg·L^−1^) was sprayed on the industrial hemp leaves in the afternoon at about three o’clock, and the hormone was sprayed on the leaves of the seedlings until the water stopped, which was sprayed every 3 days for three times, and the control group was sprayed with distilled water. Each 40 plants were one group and three groups were repeated. When the sex of the plants could be recognized, the number of male and female plants was counted, and the flowers and leaves were harvested for the subsequent determination of CBD content and physiological indicators. Finally, the 6-BA 60 mg·L^−1^ treated female flowers of industrial hemp were screened for optimal feminization, so the 6-BA 60 mg·L^−1^ treated female flowers of industrial hemp were collected as the treatment group, and the female flowers of industrial hemp sprayed with distilled water were collected as the control group, which were used for the subsequent sequencing of the qRT-PCR validation, transcriptome, proteome, and metabolome.

### Determination of CBD content and physiological indicators in industrial hemp

The gender of industrial hemp with different treatment methods was counted, and the best treatment method was selected. The industrial hemp in the treatment group was cleaned and dried in a 65°C oven until the sample was completely dried and taken out. Grinding 0.3 g of dried sample into powder, adding 3 mL of methanol for full fusion, standing for 10 min, filtering with 0.02 μm filter membrane, filtering twice, and changing the filter membrane once in the middle. Dilute the concentration of the filtrate to below 4 g·L^-1^, and put the diluted filtrate into a chromatographic bottle to obtain a sample solution. The sample solution was sent to Heilongjiang Industrial Hemp and Products Quality Supervision and Inspection Center for CBD content detection, and the content was detected by HPLC. The parameters were set as follows: the mobile phase was acetonitrile:0.1% phosphoric acid aqueous solution (55:45), the flow rate was 1.0 mL/min, the column temperature was 30°C and the detection wavelength was 228 nm. The SOD activity was determined by the nitrogen blue tetrazole reduction method, the CAT activity by the UV-absorbance method, the content of soluble sugars by the anthrone colorimetry method, and the content of soluble proteins by the Coomassie Brilliant Blue G-250 staining method. General parametric analyses were performed using IBM SPSS Statistics 27.0.1 software. Differences between samples were determined by one-way analysis of variance (ANOVA) and significant differences were calculated by the least significant difference (LSD) test at *P* < .05.

### *Transcriptomic analysis and qRT-PCR* id*entification of differentially expressed genes*

The 6-BA 60 mg·L^-1^ treated female flowers of hemp screened in the previous experiment were used as experimental materials and six mixed samples were randomly selected from each group of the treatment group and the control group as a biological replication, which was repeated in three times, and the samples was flash-frozen with the liquid nitrogen in a −80 °C refrigerator for subsequent sequencing and qRT-PCR experiments. The RNA extraction and cDNA library construction of the test samples were carried out according to the standard procedures of Beijing Novozymes Technology Co., Ltd., and all cDNA libraries were sequenced on the NovoMagic self-developed platform, and the results of sequencing transformed the raw image data into raw sequence data, which were filtered to obtain high-quality data. Sequence comparison between clean Reads and reference genomes was performed using STAR (http://github.com/alexdobin/STAR/), a specialized comparison software for RNA-seq. FPKM (Fragments Per Kilobase of transcript per Million fragments mapped) was utilized as an indicator of gene expression level. Differentially expressed genes (DEGs) were obtained by comparing the gene expression of the 6-BA 60 mg-L-1 treatment group with that of the control group using DESeq2 software (1.20.0). After homogenising the expression of the genes to FPKM values, *P*-value and corrected *P*-value (false discovery rate (FDR) were calculated, and fold change ≥ 2 and FDR < 0.05 were used as screening criteria for this study. Kyoto Encyclopedia of Genes and Genomes (KEGG) pathway enrichment analysis and Gene Ontology (GO) enrichment analysis of DEGs were realized by clusterProfiler (3.8.1) software, and *P*-value < .05 was screened for significantly enriched pathways. Functional characterization of DEGs was performed using Swiss-prot (http://web.expasy.org/docs/swiss-prot_guideline.html), Pfam (http://pfam.xfam.org/), GO (http://geneontology.org/), and KEGG (http://genome.jp/kegg/) databases. Twelve DEGs were randomly selected from the transcriptome data for qRT-PCR validation using an internal reference gene, GADPH, as a control for data normalization to correct for quantitative differences in cDNAs used as templates and three biological replicates were performed for each gene, and data processing was performed by the 2^-∆∆CT^ method, general parametric analyses were performed using IBM SPSS Statistics 27.0.1 software. Differences between samples were determined by one-way ANOVA and significant differences were calculated by the LSD test at *P* < .05. The correlation between RNA-Seq and q-PCR was analyzed using the Pearson correlation coefficient method with SPSS software.

### Total protein extraction and enzymatic hydrolysis

Total protein of the female flower of industrial hemp was extracted using the Bradford protein quantification kit, different concentration gradients of BSA standard protein was prepared and mixed with different dilutions of the protein solution to be tested and added to the 96-well plate, filling up the volume to 20 µL, each treatment was repeated three times, 180 µL G250 staining solution was quickly added, the absorbance value was determined at 595 nm, and the absorbance of the standard protein solution was used to plot the standard curve and calculate the protein concentration of the samples to be tested. The protein samples were subjected to SDS-PAGE gel electrophoresis, and then stained with Coomassie brilliant blue R-250, and decolorized until the bands were clear. Protein samples were added to the protease solution, trypsin, and 100 mM TEAB buffer, mixed and digested at 37°C for 4 h, followed by trypsin and CaCl_2_ digestion overnight. Formic acid was added to make pH < 3, mixed well, and centrifuged at room temperature and 12,000 g for 5 min, the supernatant was taken and slowly passed through a C18 desalting column, after which it was washed three times using a washing solution (0.1% formic acid, 3% acetonitrile), and then an appropriate amount of eluent (0.1% formic acid, 70% acetonitrile) was added, the filtrate was collected and lyophilized.

### Protein liquid quality detection and data analysis

Mobile phase A (100% water, 0.1% formic acid) was prepared, 10 µL of liquid A was used to dissolve the lyophilized powder, centrifugation was performed at 4°C, 14,000 g for 20 min, and 1 µg of the supernatant was injected into the sample, and the sample was analyzed by using the EASY-nLC TM 1200 nano scale-up UHPLC system, the Orbitrap Exploris 480 Mass Spectrometer, the Nanospray Flex™ (ESI) ion source, the mass spectrometry was performed in a data-dependent acquisition mode, with a primary mass resolution of 60,000 (200 m/z) and a maximum C-trap capacity of 3 × 106, and a secondary mass resolution of 15,000 (200 m/z) and a maximum C-trap capacity of 7.5 × 104, to generate the mass spectrometry detection raw data. Protein qualitative and quantitative analyses were performed with the software Proteome Discoverer 2.5 and Spectronaut 16, and data were quality controlled with the software Python-3.5.0, spectral peptides (PSMs) with a confidence level of 99% or higher were considered credible PSMs, proteins containing at least one unique peptide were considered credible proteins for FDR validation, and peptides and proteins with FDR greater than 1% were removed. Differentially expressed proteins were identified using the *T*-test, and *P* value was calculated using the significant A algorithm, with *P*-value < .05 as the significance indicator. blast-2.2.26 was then used to combine GO (http://geneontology.org/) and KEGG (http://genome.jp/kegg/) databases for functional annotation and enrichment analysis of proteins.

### *Metabolite extraction and* id*entification*

100 mg of industrial hemp sample was placed in EP tubes for grinding with liquid nitrogen and was added 500 µl of 80% methanol solution. The sample was vortexed and shaken, allowed to stand on an ice bath for 5 min, and centrifuged at 15,000 g at 4°C for 20 min; a certain amount of supernatant was diluted with mass spectrometry-grade water when methanol content reached 53%; the supernatant was centrifuged at 15,000 g at 4°C for 20 min, and the supernatant was collected, injected, and analyzed using LC-MS. Peak area correction was performed with the first QC, and the peak area was also quantified and compared with the mzCloud, mzVault, and Masslist databases. The data were transformed using metaX and subjected to principal component analysis (PCA) and partial least squares discriminant analysis (PLS-DA), which led to the VIP values for each metabolite. For the univariate analysis part, a *t*-test was used to calculate the statistical significance (*P*-value) of each metabolite between the two groups and the multiplicity of difference (FC) of metabolites between the two groups. The default criteria for differential metabolite screening were VIP > 1, *P* < .05, FC ≥ 2, or FC ≤ 0.5.

## Results

### Determination of sex statistics, CBD content, and physiological indices of industrial hemp under different concentrations of 6-BA treatment

**Figure 1.**
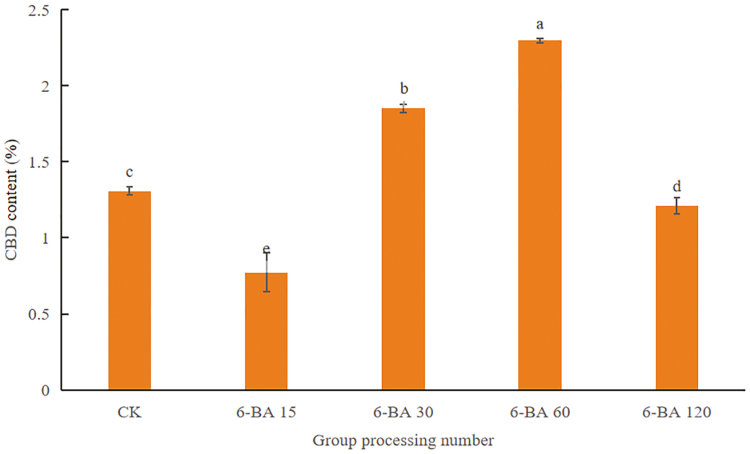
Effect of different concentrations of 6-BA on CBD content in female flowers of industrial hemp. Different letters represent significant differences.

**Figure 2.**
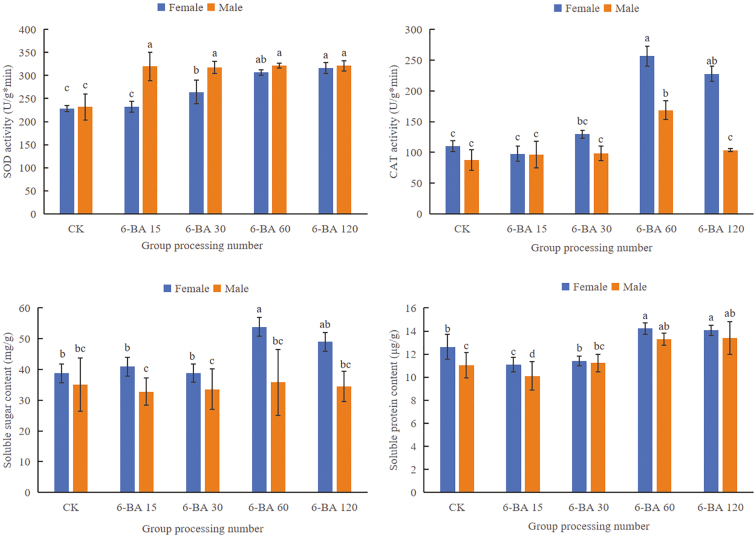
Effects of different concentrations of 6-BA on SOD and CAT activities, soluble sugar, and soluble protein contents of male and female leaves of industrial hemp. Different letters represent significant differences.

The number of female plants and CBD content of industrial hemp peaked at 6-BA 60 mg·L^−1^ treatment, and the THC content was lower than 0.3%, at which time the number of female plants and CBD content increased by 43% and 0.44% (dry weight), respectively, compared with the control (**Table 1**, **Fig. 1**). After 6-BA treatment, industrial hemp SOD activity was significantly higher than the control (*P < *.05), and the SOD activity of female hemp was lower than that of male hemp; CAT activity, soluble sugar, and soluble protein content of female hemp were higher than that of male hemp, and with the increase of 6-BA concentration, the activity and content of the above three substances showed a tendency to increase and then decrease in both female and male hemp, and all of them reached the peak at 6-BA 60 mg·L^−1^ (**Fig. 2**). In conclusion, 6-BA helped to increase the ratio of female to male in industrial hemp, especially the most significant effect in 6-BA 60 mg·L^−1^ treatment group.

**Table 1.** Sex statistics of industrial hemp under different concentrations of 6-BA treatment.

	Concentration (mg·L^−1^)	Number of female plants	Number of male plants
CK	0	19.67 ± 3.06f	20.33 ± 3.06f
6-BA	15	27.00 ± 3.00c	13.00 ± 3.00k
	30	28.00 ± 2.00bc	12.00 ± 2.00k
	60	36.00 ± 2.65a	4.00 ± 2.65o
	120	30.67 ± 9.29b	9.33 ± 9.29m

Note: Different letters represent significant differences.

### Screening, annotation, and functional enrichment analysis of industrial hemp DEGs after 6-BA treatment

**Figure 3.**
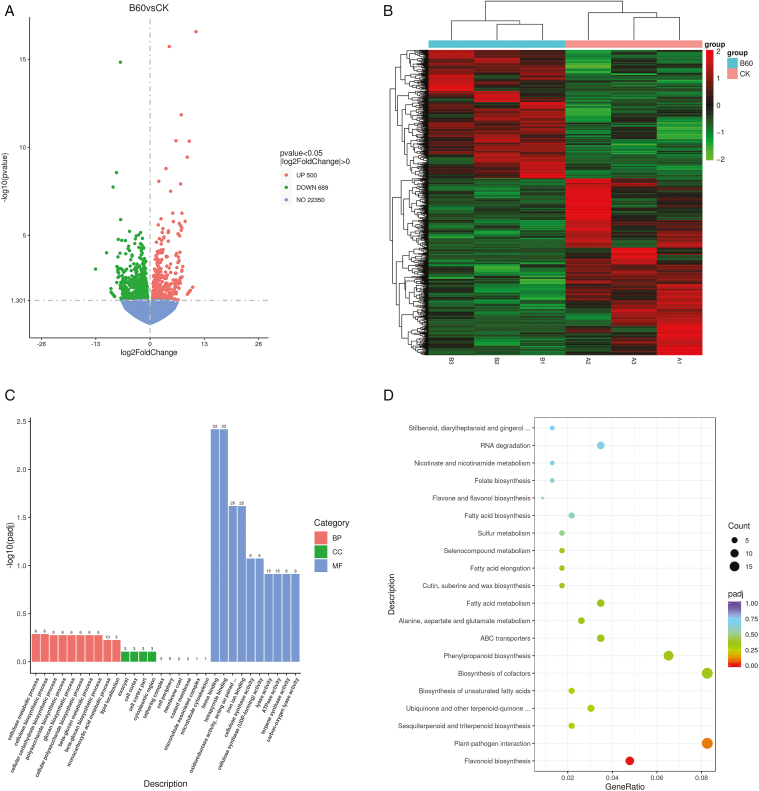
Transcriptome analysis of female flowers of industrial hemp under 6-BA treatment. **(A)** DEGs volcano map. Up-regulated genes are indicated by red dots, down-regulated genes are indicated by green dots, and the blue dashed line indicates the threshold line for DEGs screening criteria. **(B)** DEGs clustering heat map. The redder the color, the higher the expression, and the greener, the lower the expression. **(C)** GO enrichment histogram. The vertical coordinate is the significance level of enrichment, and different colors indicate different functional classifications. **(D)** DEGs KEGG enrichment bubble diagram. The size of the dots represents the number of genes annotated to the KEGG pathway, and the color from red to purple represents the significance of enrichment from large to small.

Comparing the genes in the 6-BA-treated group (BA) with the control group (Ctrl), a total of 1189 DEGs were screened, of which 500 genes were up-regulated for expression and 689 genes were down-regulated for expression (**Fig. 3A**). Cluster analysis of DEGs with the same expression patterns in the 6-BA-treated and control groups (**Fig. 3B**) showed that the expression patterns of the genes in the two groups were different, suggesting that there may be opposing mechanisms of action between these genes in hemp female development.

**Figure 4.**
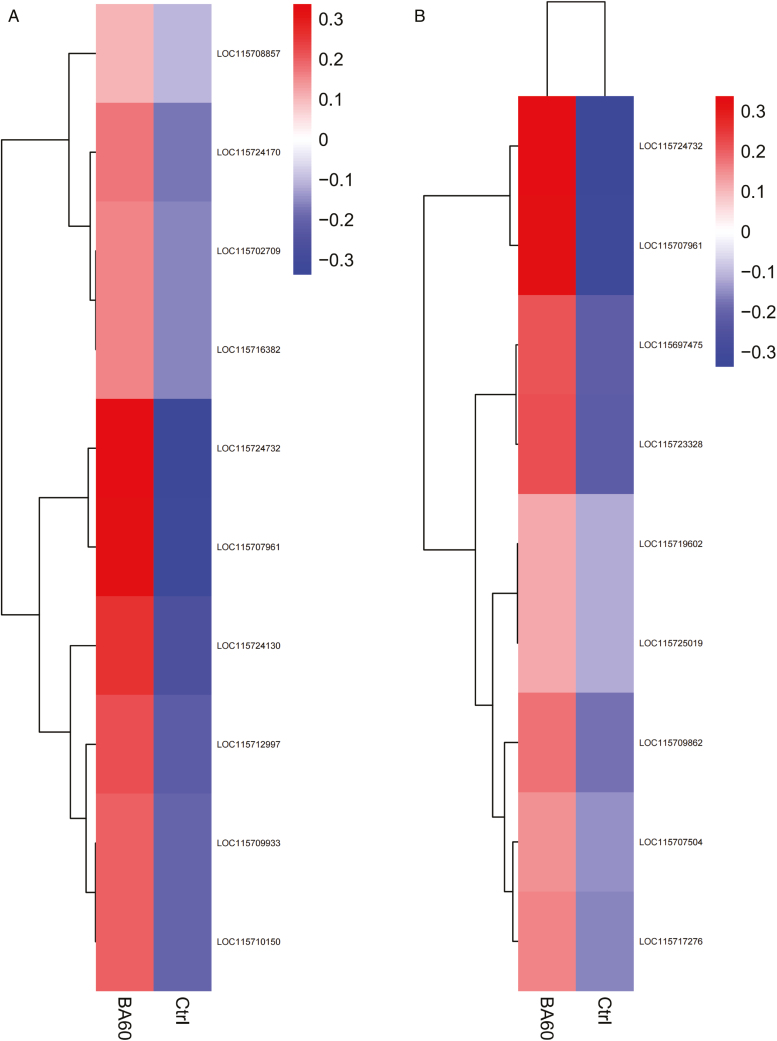
**(A)** Cluster analysis of 10 up-regulated genes in flavonoid biosynthesis. **(B)** Cluster analysis of nine up-regulated genes in phenylpropane biosynthesis.

The biological functions of these DEGs were categorized using GO enrichment analysis (**Fig. 3C**), and a total of 593 GO terms were enriched in biological processes (BPs), cellular components (CCs), and molecular functions (MFs), with numbers of 321, 59, and 213, respectively. Cellulose metabolism process, cellulose synthesis process, and cellular carbohydrate synthesis process were the most enriched categories in BPs, suggesting that saccharides may provide some nutritional supplements for the development of hemp female flowers, exocysts, cell cortex, and cytoplasmic region were the most enriched categories in CCs, and the most enriched categories in MFs were hemoglobin-binding, tetrapyrrole-binding, and ferric iron ion-binding. To further investigate the potential function of these DEGs in the feminization of industrial hemp by 6-BA, KEGG pathway enrichment analysis of DEGs was performed, and a total of 382 DEGs were assigned to 98 KEGG pathways, in which biosynthesis of secondary metabolites was significantly enriched (*P* < .05), **Fig. 3D** showed the 20 metabolic pathways involved in the highest number of DEGs in the response of industrial hemp female flowers to 6-BA, with the most DEGs involved in flavonoid biosynthesis and phenylpropane biosynthesis involved in the highest number of DEGs, 11 and 15, respectively. The 10 up-regulated genes in flavonoid biosynthesis were clustered and analyzed (**Fig. 4A**), and gene expression was up-regulated in all of them compared with the control, and two representative genes (LOC115709933, LOC115708857) were annotated as *CYP450* (flavonoid 3′-monooxygenase) and *FLS* (flavonol synthase). Cluster analysis of the nine up-regulated genes in phenylpropane biosynthesis (**Fig. 4B**) also revealed two representative genes (LOC115725019, LOC115709862), annotated as *CCL1* (4-coumarate-CoA ligase) and *PAL1* (phenylalanine ammonia-lyase 1), and these genes may work together to regulate the process of female flower differentiation in hemp. From the above, it is hypothesized that transcriptional regulation occurring in the cell cortex plays an important role in the female flower regulatory pathway of industrial hemp, in which heme- and iron-containing genes are more important, in addition to flavonoid biosynthesis and phenylpropane biosynthesis, which are also closely related to estrogenization.

### qRT-PCR validation of differentially expressed genes

**Figure 5.**
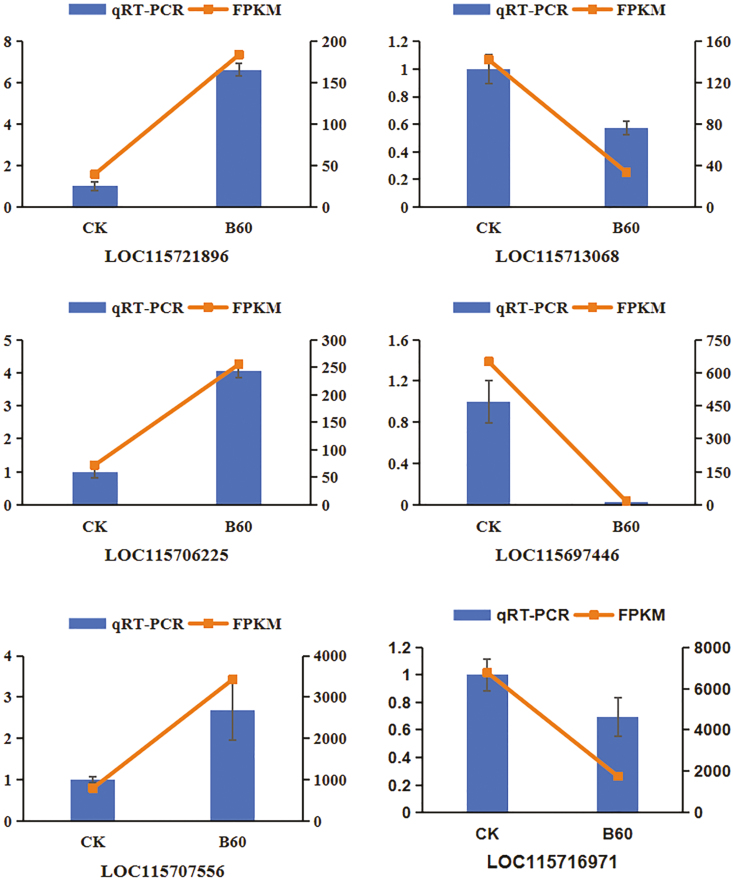

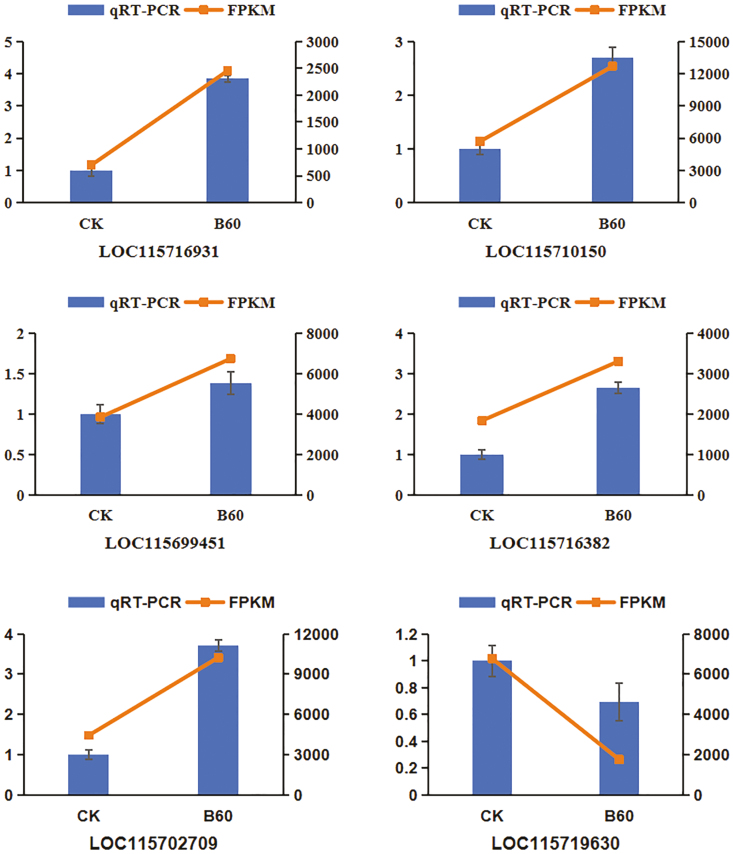
qRT-PCR detection of gene expression patterns in industrial hemp after 6-BA treatment. The left y-axis represents the relative expression level, and the right y-axis represents the FPKM value. The bar chart represents the mean ± SE.

There was a good agreement between the analysis results of qRT-PCR (**Fig. 5**) and the expression levels of 12 genes detected by RNA-seq, The correlation coefficient between the two expression measures was 0.8129 (*R*^2^ = 0.8129), and the difference was significant (*P < *.05) compared with the control group, proving that the transcriptome sequencing results had high accuracy and confidence, and 6-BA played a certain regulatory role in the sex differentiation of industrial hemp.

### Proteomic analysis of DEPs in female flowers of industrial hemp under 6-BA treatment

**Figure 6.**
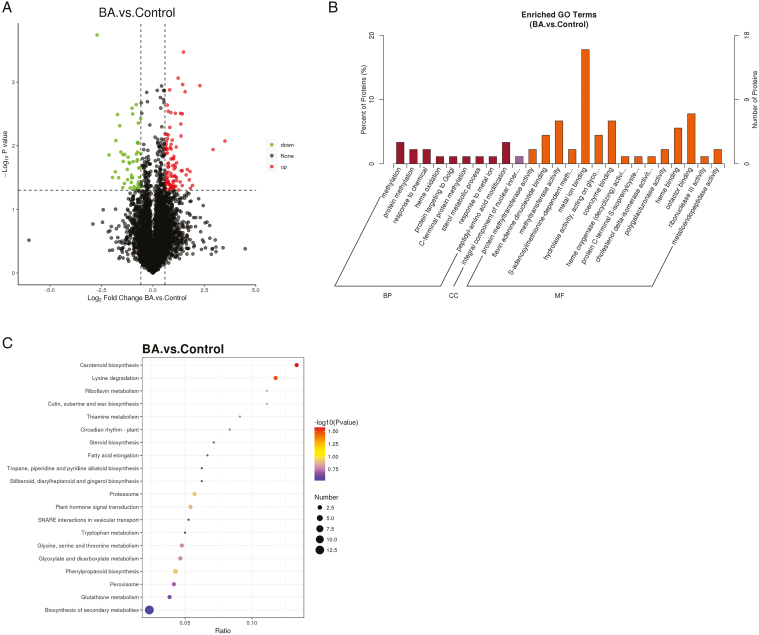
Proteomic analysis of female flowers of industrial hemp under 6-BA treatment. **(A)** DEPs volcano map. black represents proteins with non-significant differences, red represents up-regulated proteins, and green represents down-regulated proteins. **(B)** GO enrichment histogram. The figure illustrates the enrichment results in the three categories (*P* value ≤ .05). **(C)** DEPs KEGG enrichment bubble diagram. The redder the color of the dot, the greater the reliability of the test, and the larger the size of the dot, the more DEPs there are within that pathway.

A total of 168 differentially expressed proteins (DEPs) were identified in the 6-BA treatment compared with the control, of which 108 were up-regulated and 60 were down-regulated (**Fig. 6A**). GO enrichment analysis of these DEPs (**Fig. 6B**) showed that DEPs in BPs were mainly enriched in the protein, methyltransferase pathway, DEPs in CCs were mainly enriched in the nucleus, and DEPs in MFs were mainly enriched in the metal ion binding pathway. Proteins usually interact with each other to perform their corresponding functions, so we also analyzed the KEGG enrichment of DEPs, which were assigned to a total of 39 KEGG pathways, and the pathways of secondary metabolites, and metabolic categories were the significantly enriched branches. (**Fig. 6C**) are the 20 pathways with the most DEPs involved in the response of industrial hemp female flowers to 6-BA, among which two metabolic pathways, phytohormone signaling, and phenylpropanoid biosynthesis, were significantly enriched and had the largest number of participating proteins. There were two significantly up-regulated DEPs in phytohormone signaling (XP_030478342.1, XP_030499936.1), which were related to auxin and cytokinin responses, respectively, and two significantly up-regulated DEPs in phenylpropanoid biosynthesis (XP_030504384.1, XP_030508485.1), both of which play important roles in phenylpropanoid biosynthesis. It is hypothesized that proteins associated with cytokinins and phenylpropanoids may play a role in the ability to regulate reproductive growth in industrial hemp plants.

### Screening and enrichment analysis of differential metabolites

**Figure 7.**
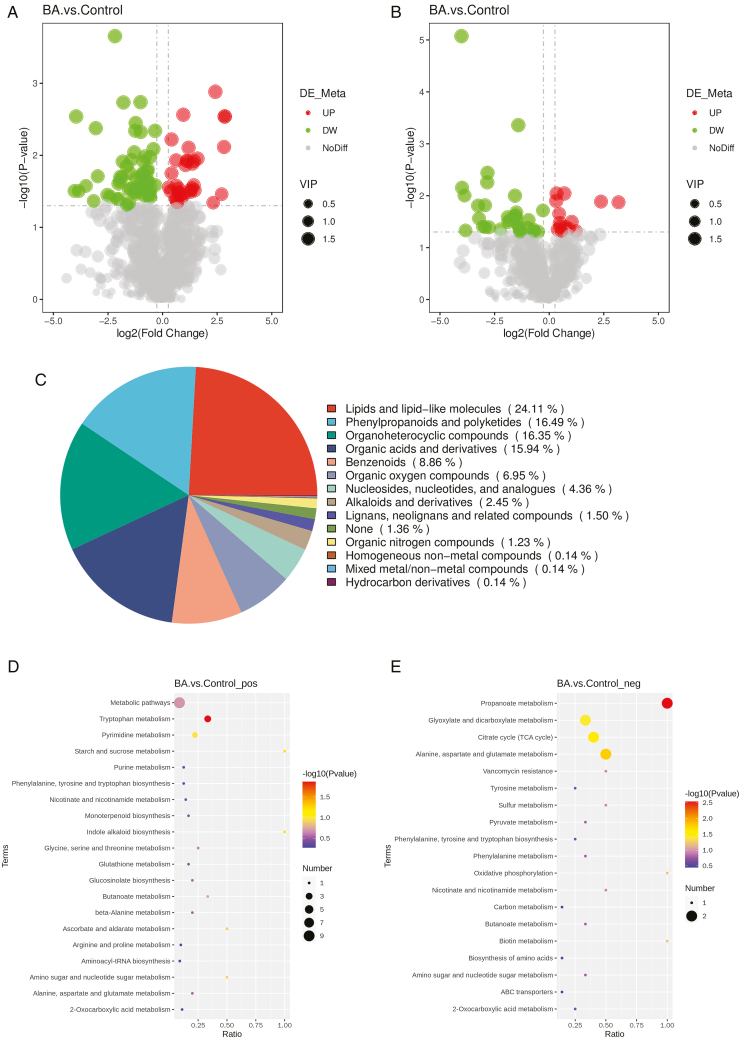
Metabolomic analysis of female flowers of industrial hemp under 6-BA treatment. **(A/B)** POS/NEG DAMs volcano map. Each point in the volcano diagram represents a metabolite, with significantly up-regulated metabolites represented by red points and significantly down-regulated metabolites represented by green points. **(C)** DAMs classification chart. **(D/E)** POS/NEG DAMs KEGG enrichment bubble diagram. The redder the color of the dot, the greater the reliability of the test, and the larger the size of the dot, the more DAMs there are within that pathway.

A total of 1525 metabolites were identified after 6-BA treatment, with 138 DAMs (differential metabolites) compared to the control, of which 48 were significantly up-regulated (34 positive ions and 14 negative ions) and 90 were significantly down-regulated (58 positive ions and 32 negative ions) (**Fig. 7A, B**). First, the identified DAMs were classified and annotated into 12 categories, the top three were: lipids and lipid-like molecules with 24.11%, phenylpropanoids and polyketides with 16.49%, and organic heterocyclic compounds with 16.35% (**Fig. 7C**).To gain insight into the changes in metabolites during the development of the female flowers of industrial hemp, KEGG enrichment analysis of these DAMs was performed. Among the enrichment pathways of these DAMs, most of the positive ions were enriched in metabolic pathways, such as metabolic pathways, tryptophan metabolism, pyrimidine metabolism, starch, and sucrose metabolism, phenylalanine, tyrosine, and tryptophan biosynthesis (**Fig. 7D**), and most of the negative ions were enriched in the pathways, such as propionic acid metabolism, glyoxylate, and dicarboxylic acid metabolism, the citric acid cycle, alanine, aspartic acid and glutamic acid metabolism, phenylalanine, tyrosine, and tryptophan biosynthesis pathways (**Fig. 7E**), with phenylalanine biosynthesis enriched in both types of metabolic pathways.

### Combined transcriptome, proteome, and metabolome analysis

**Figure 8.**
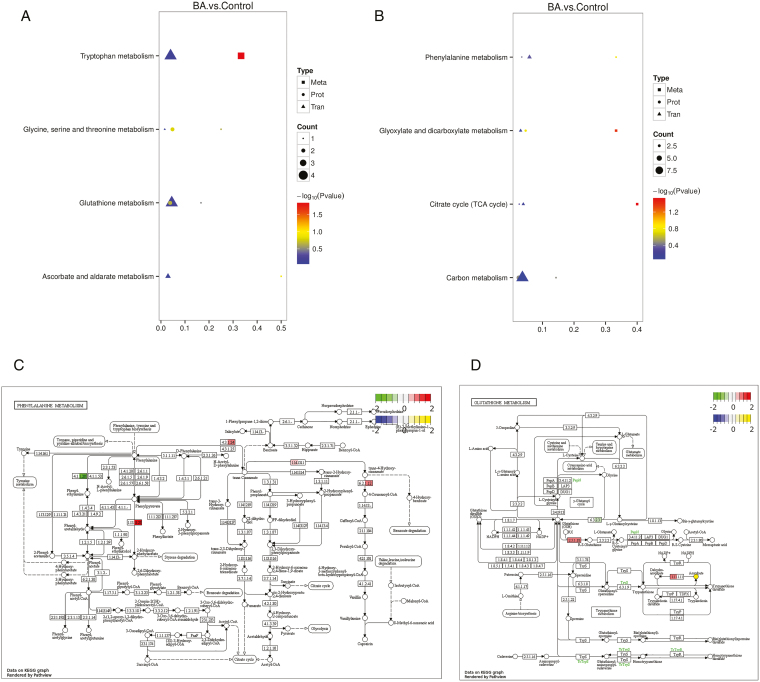
Combined histological analysis. **(A/B)** POS/NEG DEGs, DEPs, and DAMs KEGG enrichment bubble diagram. The horizontal coordinate is the ratio (Ratio) of the number of DAMs, DEPs, or DEGs enriched to that pathway to the number of metabolites, proteins, or genes annotated to that pathway, and the vertical coordinate is the metabolome-proteome-transcriptome co-enrichment to the KEGG pathway. **(C)** Biosynthesis of phenylpropane pathview enrichment pathway map. **(D)** Glutathione metabolism pathview enrichment pathway map. Circles represent metabolites, blue circles are down-regulated differential metabolites and yellow circles are up-regulated differential metabolites; the left half of the box represents genes and the right half represents proteins, where green represents genes differentially down-regulated in expression and red represents genes differentially up-regulated in expression. The log2(FC) of metabolites and genes are used as markers, with the first legend green to red representing the FC of genes and the second legend blue to yellow representing the FC of metabolites.

All the obtained DEGs, DEPs, and DAMs were mapped to the KEGG pathway database separately to obtain common pathway information and identify the major biochemical pathways and signaling pathways in which they are jointly involved. Positive ions were enriched in tryptophan metabolism, glycine, serine, and threonine metabolism, glutathione metabolism, ascorbic acid, and aldolate metabolism pathways (**Fig. 8A**), while negative ions were enriched in phenylalanine metabolism, glyoxylate, and dicarboxylic acid metabolism, citric acid cycle, and carbon metabolism pathways (**Fig. 8B**).

The phenylpropane biosynthetic pathway is essential for plant survival, and the pathway provides plants with precursors for a large number of secondary metabolites(Hamberger and Hahlbrock 2004), such as coumarins, flavonoids, and lignans, all of which belong to the phenylpropane class of compounds. Therefore, the phenylpropane metabolic pathway was analyzed (**Fig. 8C**), in which three genes (LOC115721908, LOC115709862, LOC115698862), one protein (XP_030504384.1), and one metabolite (succinic acid) were significantly up-regulated in the pathway, and the significantly up-regulated genes and proteins were also identified in the transcriptome and proteome. The glutathione metabolic pathway (**Fig. 8D**) was also analyzed, and a total of four genes (LOC115714913, LOC115704197, LOC115724756, LOC115719440), two proteins (XP_030487766.1, XP_030509992.1), and one metabolite (Vitamin C) were significantly altered, and these differentially altered genes, proteins, and metabolites were associated with oxidoreductases. It is hypothesized that the phenylpropane and glutathione metabolic pathways may be related to female flower differentiation in industrial hemp.

## Discussion

### Cytokinins may induce female flower differentiation in industrial hemp

Phytohormones play an important regulatory role in the process of plant sex differentiation and are one of the inducible signals for plant flower sex differentiation (Airoldi 2010), among which cytokinins are important components of phytohormones, which are involved in several processes, such as plant nutrient growth, male gametophyte development and female gametophyte development (Cortleven et al. 2019). For example, *ARR8* and *ARR12* in the cytokinin signaling pathway of cassava flowers were significantly up-regulated in female flowers, and the cytokinin content of female flowers was significantly higher than that of male flowers, and cytokinin may have a promotional effect on the differentiation of female flowers of cassava (Chen et al. 2023). High levels of ZR in persimmon could promote the differentiation of male flower buds by promoting the development of gynoecium protoplasts, which leads to female unisexual flowers (Sun et al. 2017). Industrial hemp belongs to dioecious plants, and previous studies have shown that in hemp, in addition to genetic factors, epigenetic mechanisms may also be related to sex differentiation (Truta et al. 2007), ecological factors can affect the balance of male and female in hemp plants, and phytohormones interact with genetic factors, thus determining the sex expression (Soldatova and Khryanin 2010), and the sex determination in industrial hemp can be altered through the use of exogenous growth regulators or chemical substances, which can affect the ratio of endogenous hormones, which in turn affects the incidence of sex organs (Malabadi et al. 2023), the above studies suggest that endogenous hormones can regulate sex in hemp. Many studies have shown that cytokinins can induce the differentiation of female flowers in hemp (Chailakhyan and Khryanin 1978; Soldatova and Khryanin 2010; Petit et al. 2020; Flajsman, Slapnik and Murovec 2021). The purpose of this experiment was to cultivate more female hemp and increase the CBD content of industrial hemp, spray treatment with 6-BA 60 mg·L^-1^ at the three-leaf stage of seedlings increased the number of females and the CBD content by 43% and 0.44%, respectively, compared with the control, and the CAT activity, soluble sugar, and soluble protein content of females were higher than that of males, which was also by the results of previous studies (Truta et al. 2002; Shi et al. 2012; DeSoto, Olano and Rozas 2016; Zhou 2021). Therefore, we hypothesized that cytokinins may induce female flower differentiation in industrial hemp. In this experiment, more DEPs were found enriched to the phytohormone signaling pathway in the pathway significantly enriched in the proteome, and two of the significantly up-regulated DEPs (XP_030478342.1, XP_030499936.1) were related to auxin and cytokinin responses, respectively, which further validated that cytokinin could induce industrial hemp the female flower differentiation.

### Phenylpropane metabolic pathways and flavonoids may be associated with female flower differentiation in industrial hemp

The enrichment of DEGs to the two metabolic pathways of flavonoid biosynthesis and phenylpropane biosynthesis was the most abundant and significantly enriched in the female flowers of industrial hemp in comparison with the control by 6-BA treatment, suggesting that these two metabolic pathways may be the important pathways affecting the differentiation of female flowers of industrial hemp. Among them, phenylpropane biosynthesis was also enriched in both proteomic and metabolomic KEGG pathway analyses, with two significantly up-regulated genes *CCL1* (LOC115725019) and *PAL1* (LOC115709862), a protein C4H (XP_030504384.1), a metabolite succinate. The phenylpropane biosynthetic pathway is essential for plant survival and was activated under the stimulation of abiotic stress factors, such as drought, heavy metals, salinity, temperature extremes, and ultraviolet radiation (Hoffmann et al. 2004; Mittler 2006; Agati et al. 2011; Fini et al. 2011). The phenylpropane metabolic pathway starts with the production of phenylalanine from the upstream glycolysis pathway and mangoxalic acid pathway together, which is later activated by phenylalanine ammonia lyase (PAL), C4H (cinnamic acid 4-hydroxylase) and 4CL (4-coumarate-CoA ligase) sequentially catalyzing the sequential reaction to produce cinnamic acid, p-hydroxycinnamic acid, and p-coumaroyl coenzyme A (Muro-Villanueva, Mao and Chapple 2019). These compounds will serve as upstream products of the phenylpropanoid metabolism reaction and will be ultimately converted to various phenylpropanoids, such as coumarins, flavonoids, and lignans, by a series of enzymes (Fraser and Chapple 2011).

*CCL1* belongs to the 4CL gene family, which is important in plant growth and development, and it acts in the last step of phenylpropane metabolism. 4CL proteins are encoded by a small gene family and exist in many different isoforms, belonging to the family of adenylate-forming enzymes, which catalyze the conversion of 4-coumaric acid salt and other hydroxycinnamates into the corresponding CoA thiohydroxylate esters (Stuible et al. 2000), which generate important natural product biosynthetic precursors such as lignins and various small phenolic compounds through a variety of these thioesters generating precursors for the biosynthesis of important natural products, such as lignin, flavonoids, and various small phenolic compounds through various branching pathways (Boudet 2000). *PAL1* belongs to the PAL gene family, which plays an important role in the first stage of the phenylpropane metabolic pathway, PAL is a key enzyme controlling the transition of the primary metabolism to the secondary metabolism, which determines whether the phenylpropane metabolic pathway can proceed smoothly (Shang, Wu and Ma 2022). C4H is not only a key enzyme in the phenylpropane metabolic pathway but also plays an important role in the biosynthesis of a wide range of plant metabolites (including fatty acids, phenylpropanoids, alkaloids, and terpenoids) (Liu et al. 2018). The expressions of *CCL1*, *PAL1,* and C4H in the female flowers of industrial hemp treated with 6-BA were significantly up-regulated, which proved that phenylpropanoids had a strong metabolic reaction, that is, phenylpropanoids were accumulated, as well as flavonoids and lignin precursors, so it was possible to enhance the rigidity of cell wall to support the morphogenesis of pistil. It is speculated that the metabolic pathway of phenylpropane may be related to the female flower differentiation of industrial hemp. Although there is no direct evidence at present, studies have shown that flavonoids are related to the gender differentiation of flowers. For example, significant enrichment of flavonoid metabolism during flower bud development in cabbage suggests that in cabbage flavonoid metabolism is closely related to the development of the floral organ (Ping 2017), flavonoids in the *Broussonetia papyrifera* leaves accumulate gradually with developmental time and are more abundant in females than in males (Jiao et al. 2022), and in transcriptomic and metabolomic analyses concerning the sex differentiation of mulberry flowers, the biosynthesis of flavonoids compounds was the pathway that was significantly enriched between female and male flowers of mulberry (Liu et al. 2022), and some researchers have found that the biosynthesis pathway of flavonoids is related to the formation of male flowers in the jatropha tree about male flower formation (Hui et al. 2017), and under limited phosphorus conditions, flavonoid biosynthesis was greater in female hickory leaves than in male hickory (Randriamanana et al. 2014).

The flavonoid metabolic pathway was also the significantly enriched pathway in the female flowers of industrial hemp in this study and two significantly up-regulated genes, *CYP450* (LOC115709933), and *FLS* (LOC115708857), were identified. Among them, *CYP450* belongs to the gene family of plant cytochrome P450 enzymes, which are involved in a variety of biosynthetic reactions in organisms, and is a class of B cytochrome superfamily proteases with heme as a cofactor, and a key enzyme in the biosynthesis of flavonoids. CYP450s are also involved in the synthesis and catabolic metabolism of phytohormones and play a crucial role in the maintenance of endogenous hormone homeostasis in plants. For example, the *CYP94* subfamily genes encode fatty acid ω-oxidases, where *CYP94B3* and *CYP94C1* encode enzymes involved in the metabolism of the jasmonyl-isoleucine complex, which affects jasmonate metabolic pathways (Koo, Cooke and Howe 2011), *AtCYP85A2* was involved in the conversion of brassinosterone to brassinolide in Arabidopsis thaliana, suggesting that *CYP85A2* is involved in the synthesis reaction of brassinolide (Nomura et al. 2005), *GmCYP78A72* from soybean regulates flower and seed development (Zhao et al. 2016). The accumulation of flavonoids enhances the sensitivity of cells to 6-BA, and the hormone synthesis pathway mediated by *CYP450* promotes the elongation of industrial hemp pistil primordial cells, and cooperates with cytokinin to complete the morphogenesis of flower organs. Therefore, it was hypothesized that the up-regulation of *CYP450* expression and further accumulation of flavonoids in female flowers of industrial hemp under 6-BA treatment induced the differentiation of female flowers of industrial hemp.

### Carbohydrates may act synergistically with cytokinins to induce female flower differentiation in industrial hemp

Another overexpressed gene in the flavonoid metabolic pathway, *FLS*, belongs to the flavonol synthase-like gene family, which converts dihydroflavonols, a product of the second stage of flavonoid biosynthesis, into various flavonoids and glycosides, whereas starch and sucrose metabolism were also enriched pathways in the transcriptome and metabolome of female flowers of hemp. in the present study. Previous studies have shown that glycolysis plays an important role in flower development and that starch and sucrose metabolism are significantly enriched pathways in the transcriptome and metabolome of mulberry flower sex differentiation, and that of the 12 DEGs associated with glycolysis, nine of them were overexpressed in female flowers, and three of them were overexpressed in male flowers (Liu et al. 2022), this pathway was also enriched between female and male flowers of spinach (Li et al. 2020). The *FLS* was also overexpressed in 6-BA-treated female flowers of industrial hemp, so we hypothesized that the enrichment of the starch and sucrose metabolic pathways may be related to the overexpression of *FLS* genes and that carbohydrates may act synergistically with cytokinins in inducing the differentiation of female flowers of industrial hemp. This is also consistent with earlier findings, for example, that in pepper, cytokinin was an important factor affecting male flower differentiation and may interact synergistically with carbohydrates to regulate reproductive growth in pepper (Hui et al. 2022). Cytokinin and glucose signaling pathways are interdependent and overlap in Arabidopsis seedlings, and glucose can affect 76% of cytokinin-regulated genes at the genome-wide level (Kushwah and Laxmi 2014), and a series of genes related to sugar metabolism and signaling were associated with higher levels of sucrose, fructose, and glucose in 6-BA-treated apple flower buds compared to untreated flower buds during flower induction (Li et al. 2019). The above findings are mainly because endogenous levels of cytokinins can regulate the expression of sucrose transporter proteins (McIntyre, Bush and Argueso 2021), and further support the reliability of the results of the present study.

### Functional analysis of genes, proteins, and metabolites related to glutathione metabolism

Glutathione is an essential metabolite with multiple functions in plants, and glutathione-related pathways play important roles in biosynthetic pathways, detoxification, antioxidant, biochemistry, and redox homeostasis (Noctor et al. 2012). The GST (glutathione S-transferase family) in plants is known for their structural and functional diversity, and their functions have been identified in a wide range of plants, such as xenobiotic detoxification, secondary metabolism, growth and development, including flower-associated development (Dixon and Edwards 2010; Noctor et al. 2012). The glutathione metabolic pathway was also the enriched pathway in this experiment at the time of combined histological analysis, in which three genes (LOC115714913, LOC115704197, LOC115724756), one protein (XP_030509992.1) with significant changes belonged to the GST family, and the other protein with significant changes (XP_030487766.1) belongs to APX (ascorbate peroxidase) and the metabolite is vitamin C. These genes, proteins, and metabolites are associated with antioxidants. Among them, APX belongs to the heme-containing peroxidase family, which catalyzes the H_2_O_2_-dependent oxidation of a wide range of organic molecules and plays a crucial role in plant growth regulation (Pandey et al. 2017), whereas vitamin C not only acts as a specific electron donor for APX and regulates the above processes at the molecular and cellular levels but also participates in different stages of plant growth and development, such as seed maturation and germination, and flowering (Paciolla et al. 2019), significant up-regulation of genes and proteins related to glutathione reductase and ascorbate peroxidase has also been found in cytokinin-sprayed ryegrass and broccoli (Liu et al. 2013; Me, Zhang and Huang 2016). Therefore, it is speculated that 6-BA treatment directly upregulates the expression of GST and APX family genes, thus improving the activity of antioxidant system, maintaining cell division and promoting the formation of female flower primordium of industrial hemp. To sum up, 6-BA can further induce the feminization of industrial hemp by regulating glutathione metabolic pathway.

## Conclusion

In this study, 6-BA 60 mg·L^−1^-treated female flowers of industrial hemp were performed by transcriptomic, proteomic, and metabolomic analyses, compared with the control, a total of 1189 DEGs, 168 DEPs, and 138 DAMs were screened, which were jointly involved in the regulatory network of female flower development of industrial hemp. Through combined histological analysis a pathway model of plant hormone signal transduction, starch and sucrose metabolism, flavonoid biosynthesis, phenylpropanoid metabolism, and glutathione metabolism jointly regulating the development of female flowers of industrial hemp was proposed, and the analysis of identified overexpressed genes, proteins, and metabolites further proved the important roles of the above pathway model in the development of female flowers in industrial hemp. All the data, candidate genes, and proteins from the combined histological analysis obtained in this study can be used as references for subsequent studies or breeding of industrial hemp and other plants in terms of sex differentiation.

## Data Availability

The multi-omics data that support the findings of this study are openly available in NCBI Sequence Read Archive (SRA) data-base at (https://www.ncbi.nlm.nih.gov) with the BioProject ID (PRJNA1240697).
